# Atlantic & Great Western R. W.

**Published:** 1865-11

**Authors:** 


### ATLANTIC & GREAT WESTERN R. W.

We have recently passed over this road from Cincinnati to New York, and must say that we found it much better in its facilities for accommodating a traveling public, than we had anticipated, notwithstanding we had heard flattering reports of it before.

The road in all its arrangements and appointments, exhibits a completeness and perfection, that has only been attained by most other roads, by a far more protracted term of experience, though the road is but just completed. The cars run very smoothly, occasioning little or none of that jostling that is so frequently experienced in rail-road riding.

The sleeping cars are about all that could be desired; every birth has a double bed, with a splendid mattress, and everything in perfect order, and night travel is about as comfortable as one can be in his own bed at home. We recommend all traveling east to try the Atlantic & Great Western, and we think it would not be readily exchanged for any other.

